# Scotch Tape Optical Vapor Sensor for Ethanol–Methanol Mixtures

**DOI:** 10.3390/s19245381

**Published:** 2019-12-06

**Authors:** Carlos Angulo Barrios

**Affiliations:** 1 Institute for Optoelectronic Systems and Microtechnology (ISOM), ETSI Telecomunicación, Universidad Politécnica de Madrid, Ciudad Universitaria s/n, 28040 Madrid, Spain; carlos.angulo.barrios@upm.es; 2 Department of Photonics and Bioengineering (TFB), ETSI Telecomunicación, Universidad Politécnica de Madrid, Ciudad Universitaria s/n, 28040 Madrid, Spain

**Keywords:** ethanol, methanol, optical sensor, scattering, Scotch tape, vapor sensor

## Abstract

A simple optical vapor sensor based on conventional Scotch adhesive tape, for analyzing ethanol–methanol mixtures, is proposed and demonstrated. The sensing signal relies on the variation of optical power transmitted through the tape, resulting from the response of the adhesive material to vapor sorption. The optical sensor exhibits high selectivity for ethanol vapor over methanol vapor. When exposed to vapors from ethanol–methanol liquid mixtures, the sensor shows a linear detection range of 0–100 vol%, and detection limits of 8.8 vol% ethanol and 17.6 vol% methanol. Repeatability, reproducibility, reversibility, and sensitivity to other volatile organic compounds are also studied.

## 1. Introduction

Ethanol and methanol are short chain alcohols widely employed in industrial products, such as solvents, anti-freezers, disinfectants, perfumes, fuels, and beverages [1]. Both are volatile organic compounds (VOCs), colorless liquids, and chemically very similar; but methanol is cheaper than ethanol. This has traditionally led to deliberate adulteration of ethanol by the addition of methanol for profit, which is an illegal activity that has harmful economic and health effects. Methanol is highly toxic and difficult to remove; human consumption of falsified alcoholic beverages resulting from denatured ethanol has produced severe cases of intoxication, and even deaths [2]. Thus, discrimination of these VOCs is not only important from an industrial perspective, but also from anti-fraud and public health points of view.

Methanol is very difficult to differentiate from other alcohols (especially ethanol) without chemical analysis. Typical alcohol analysis methods rely on long separation steps employing chromatography [3,4] or electrophoresis [5]. These techniques are time-consuming and require expensive equipment and qualified personnel. To overcome these drawbacks, there have been recent relevant demonstrations of chemical sensors targeting the analysis of ethanol–methanol mixtures in a cost and time effective form. In this regard, Bucur et al. [6] reported an electrochemical biosensor using enzymes (alcohol dehydrogenase and alcohol oxidase) as sensing elements, and screen printed electrodes as transducers. A chemiresistor based on Pd-polyaniline nanocomposite as a selective methanol sensor was reported by Athawale et al. [7]. Kieser et al. [8] employed a surface plasmon resonance (SPR) technique to analyze methanol–ethanol mixtures in vapor phase. Fluorescence sensors based on axially chiral unnatural triazolyl aromatic amino acid scaffolds have been also demonstrated for discrimination of methanol from ethanol, in both liquid and vapor phase mixtures [9]. Chang et al. [10] reported a methanol/ethanol colorimetric sensor based on a cholesteric liquid crystal. In addition, although not dealing directly with ethanol–methanol mixtures, there are related works on VOC sensors that demonstrate detection of pure ethanol and pure methanol vapors by means of a variety of sensing materials (typically polymers that swell in response to vapor sorption [11,12,13,14,15]) and transduction methods, such as electro-acoustic devices [11,12,13,14], electrical resistors [14,15], and optical fibers [16].

All the aforementioned chemical sensors required the synthesis of specific sensitive materials and/or specially designed transducers, which increases cost and reduces versatility. In this work, it is demonstrated that inexpensive and easily affordable Scotch tape can be used as a VOC optical sensor to distinguish between ethanol and methanol vapors. The sensitive element of the sensor is the adhesive material of the tape, which is a polymer whose morphology changes in response to vapor exposure. This change affects the amount of light scattered by the tape when it is illuminated. Thus, attenuation of directly transmitted light can be used as the sensor response. Ethanol–methanol selectivity, sensitivity, reversibility, repeatability, reproducibility, and response to other VOCs are analyzed.

## 2. Materials and Methods

**Figure 1:**
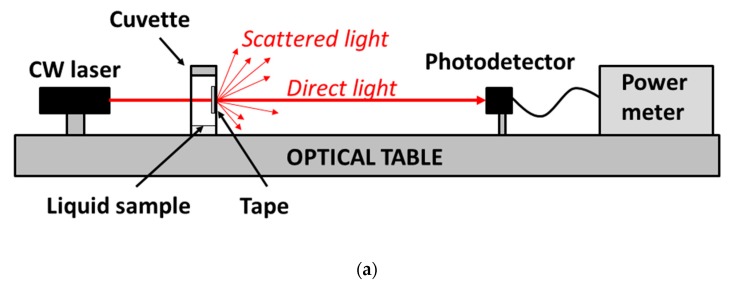

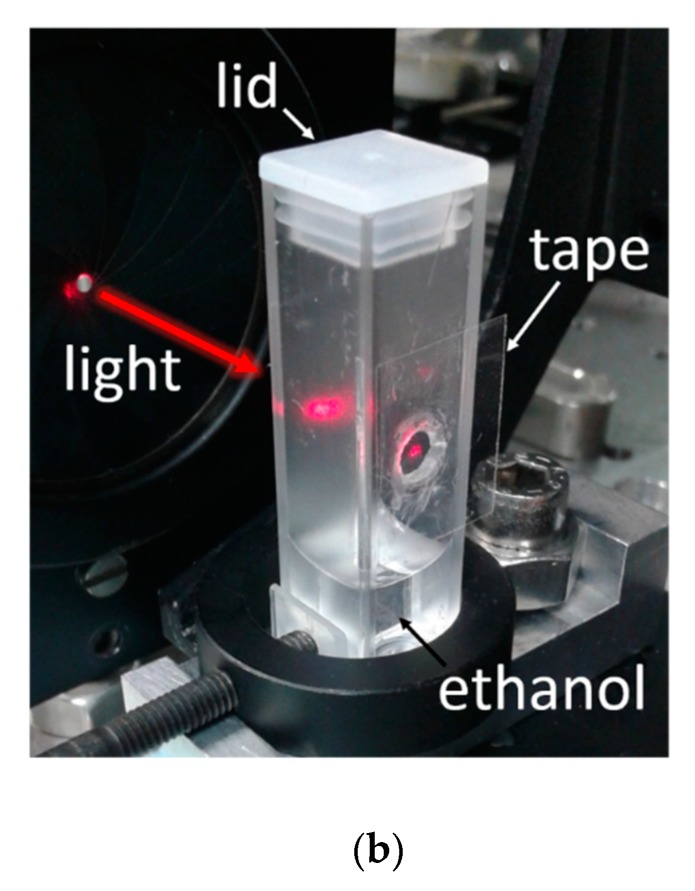
(**a**) Schematic diagram of the set-up used for optical interrogation of the Scotch tape vapor sensor. (**b**) Photograph of the cuvette containing the tape under test and a liquid sample. A 635 nm wavelength laser beam impinges the adhesive side of the Scotch tape exposed to vapor from 1 mL of liquid ethanol inside the cuvette. Direct light power transmitted through the tape is used as the sensor signal.

The Scotch tape investigated was #550 Scotch^®^ (3M, St. Paul, MN, USA), which is a general-purpose transparent adhesive tape consisting of a 20 μm thick synthetic acrylic adhesive material and a 30 μm thick bioriented polypropylene backing (from product data sheet). A transparent plastic cuvette (1 cm × 1 cm × 4.5 cm) was used as a vapor chamber for optically interrogating the adhesive side of a piece (approximately 1.5 cm × 2 cm) of Scotch tape, while exposed to vapors. For that, an ≈5 mm diameter hole was perforated on a sidewall of the cuvette, and then sealed on the outside by sticking the piece of Scotch tape under test on it. A 635 nm wavelength continuous wave (CW) laser beam (World StarTech TECRL-635) impinged the vapor-exposed adhesive tape region through the cuvette. The spot size illuminating the tape was approximately 2 mm in diameter. Directly transmitted light was collected by a photodetector (Newport 918D-SL-OD3), placed at 55 cm from the adhesive tape, and measured with a power meter (Newport 2931-C). Sensor response (i.e., transmitted light power) to vapor exposure was recorded by depositing 1 mL of liquid VOC inside the cuvette and closing it with a sealing lid to allow the evaporated VOC to interact with the adhesive tape material. Figure 1 shows a photograph of the cuvette–tape set-up, containing 1 mL of ethanol and being illuminated by the red laser beam. Sensor response to vapor exhaust was obtained by removing the cuvette lid. VOCs used in the experiments were ethanol, methanol, acetic acid, isopropanol, and ethanol–methanol mixtures. Spectral transmittance measurements were performed in the described cuvette set-up, by impinging white light from a fiber patch cable connected to a tungsten-halogen lamp into the vapor-exposed adhesive tape region, and collecting the transmitted light with another optical patch cable connected to a spectrometer (CCS200 Thorlabs). All measurements were carried out under laboratory environmental conditions: 21 °C temperature, and 20% relative humidity.

## 3. Results

**Figure 2:**
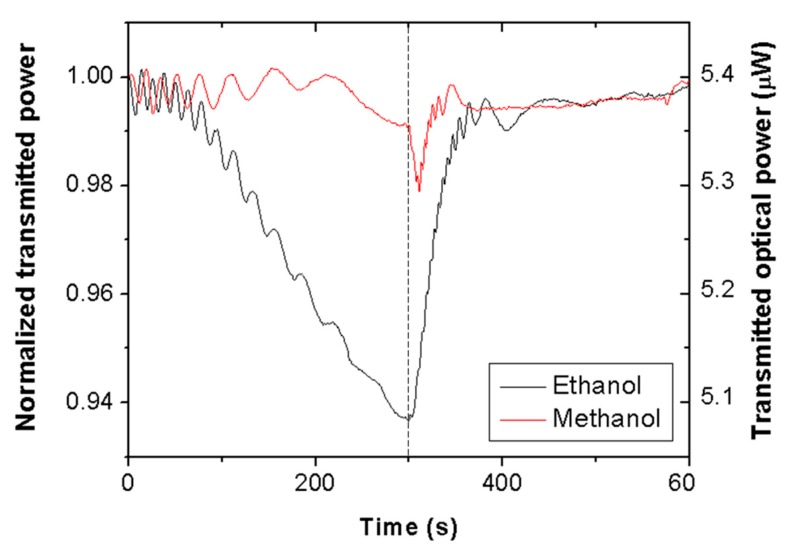
Normalized and absolute transmitted optical power at 635 nm wavelength through a piece of Scotch tape subjected to ethanol (black line) and methanol (red line) vapor exposure–exhaust measurements. At time = 0 s, the interrogation cuvette containing 1 mL of liquid alcohol was closed with a sealing lid; at time = 300 s, the lid was removed.

Figure 2 shows the time evolution of the transmitted optical power, normalized with respect to the initial value, at 635 nm wavelength through a piece of Scotch tape for ethanol (black line) and methanol (red line) vapors. At time = 0 s, the cuvette containing liquid alcohol was closed with the lid; at time = 300 s, the lid was removed. It is seen that vapor exposure produces oscillating signals, whose amplitudes and frequencies decrease with time for both VOCs. Normal transmission (T) through the tape film can be expressed as [17]:

(1) $$T=\frac{\left(1-R_{1}\right)\left(1-R_{2}\right)}{\left(1-R_{1}\right)\left(1-R_{2}\right)\;+\;4\sqrt{R_{1}R_{2}}\cdot sin^{2}\left(\frac{2\pi Ln}{\lambda }\right)}$$

where R_1_ and R_2_ are the reflection coefficients of the backing and adhesive sides of the tape, respectively; L is the tape thickness; n is the refractive index of the tape; and λ is the interrogation wavelength. Acrylic polymers tend to swell in organic solvents [18]. Thus, according to Equation (1), the observed oscillations could be attributed to the thickness variation of the tape produced by vapor swelling of the adhesive layer. In particular, taking λ = 635 nm and assuming n = 1.45 and L = (d + L_0_), where L_0_ = 50 μm is the unexposed tape thickness and d is the thickness increment due to swelling, each transmittance period would correspond to a thickness increment of d = 220 nm. Note that ethanol exposure leads to a greater number of periods than methanol exposure, meaning a thicker swollen layer for the former.

Besides the oscillation frequency, there is a clear and determining difference between the ethanol and methanol exposure signals: The former exhibits a pronounced decrease of the net transmitted power. This is an indication of high selectivity for ethanol vapor over methanol vapor. Such an effect cannot be attributed to a higher concentration of ethanol vapor in the chamber, since the evaporation rate of methanol is around twice greater than that of ethanol [19]. Sensor selectivity could then be due to a higher molecular affinity of the sensing material for ethanol, suggesting that the solubility parameter of the polymer is closer to that of ethanol (26 Mpa^0.5^ [10]) than that of methanol (29 Mpa^0.5^ [10]). A reason for the observed power decrease due to ethanol exposure might be attributed to an increment of light scattering produced by a swollen adhesive layer. The thicker the swollen layer, the larger the scattering effect, since the number of scattering centers (material and surface inhomogeneities) are expected to increase.

When the cuvette is opened, vapors are removed, and the transmitted power signals recover their initial (unexposed tape) values in approximately 1 min with negligible hysteresis. Sensor reversibility reveals effective vapor desorption (polymer deswelling), which is a characteristic behavior of physisorbed vapor molecules (as opposed to vapor chemisorption, which typically leads to no desorption). Note also, that the tape response to methanol shows a sharp dip just after opening the cuvette. This is another differential feature between ethanol and methanol responses that could be used to distinguish them. The origin of such an attenuation feature is not understood at present; it is expected that insight into this issue will be gained in future research by studying the response of other types of adhesive tape to methanol vapor exposure.

**Figure 3:**
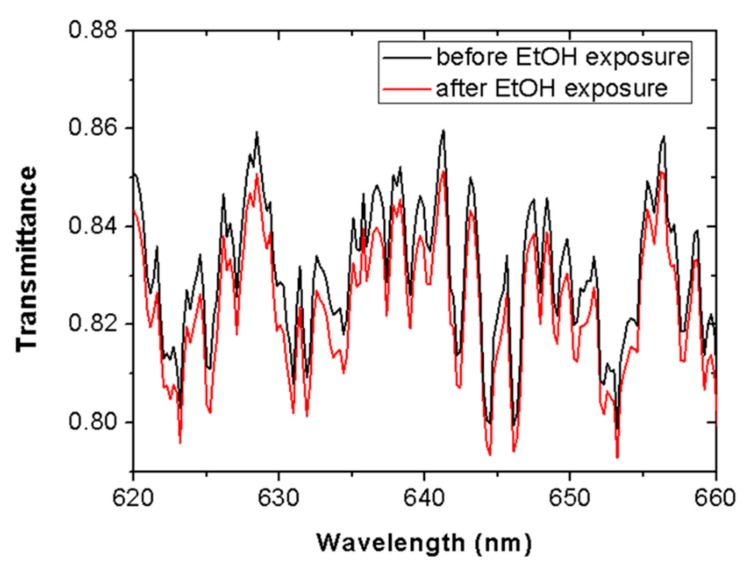
Spectral transmittance of a Scotch tape before (black line) and after (red line) 5 min of ethanol (EtOH) vapor exposure. Transmittance is attenuated in the whole spectral range due to tape–vapor interaction.

Figure 3 shows the spectral transmittance in a wavelength range around 635 nm (620–660 nm) before (black line) and after (red line) 5 min of ethanol vapor contact. It was seen that all spectral components were attenuated by a similar factor after vapor exposure. This response dismisses material absorption as a possible cause for the measured light attenuation and, therefore, it points to light scattering as the responsible process.

It is observed in the set-up of Figure 1 that, besides the tape under test, the interrogating laser beam crosses a cuvette side (that opposite to the tape) whose inner surface is also exposed to vapor; therefore, it is necessary to determine the effect of the plastic cuvette on the transmitted optical signal. For that, analogous experiments, as described before, were performed using a non-modified cuvette (i.e., without a perforated hole). To account for light scattering produced by the adhesive layer in the primary set-up (i.e., that shown in Figure 1), the laser beam was allowed to cross a piece of Scotch tape placed just outside the cuvette. In these conditions, ethanol and methanol vapor exposures of the inner surfaces of the cuvette did not lead to significant variation in the transmitted power. This indicates a negligible contribution of the cuvette to the measured signals shown in Figure 2, which can therefore be attributed entirely to the adhesive tape behavior.

**Figure 4:**
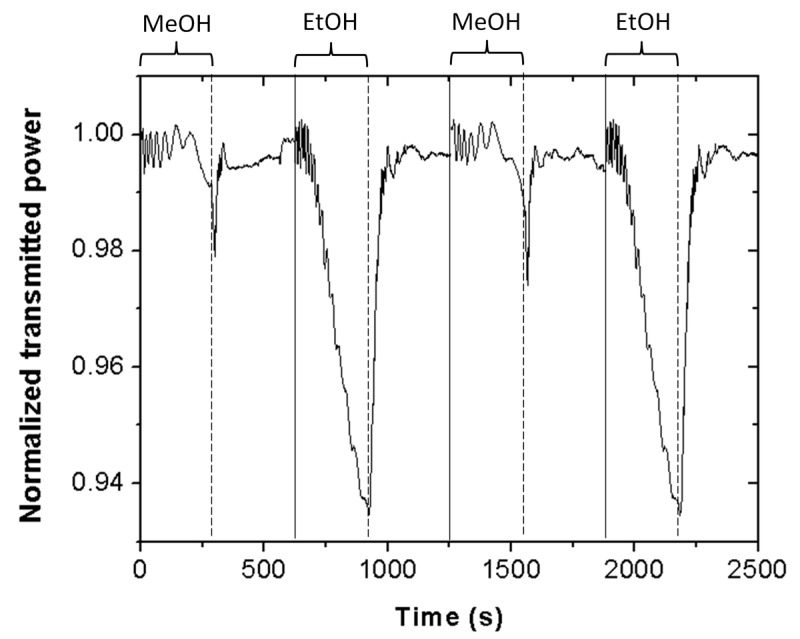
Temporal optical response of a piece of Scotch tape subjected to consecutive, alternated methanol (MeOH) and ethanol (EtOH) vapor exposure–exhaust measurements. Good repeatability is observed.

Figure 4 shows the Scotch tape sensor normalized optical transmitted power at 635 nm wavelength for a series of alternated exposure–exhaust measurements of methanol and ethanol. Vapor exposure time equals 5 min. The resulting temporal response indicates good repeatability. In particular, the standard deviations of the transmitted power at 5 min of vapor exposure for a series of three consecutive measurements were 0.2% and 0.1%, for ethanol and methanol vapors, respectively. These results demonstrate that the proposed Scotch tape sensor can reliably distinguish between ethanol and methanol vapors in only 5 min.

**Figure 5:**
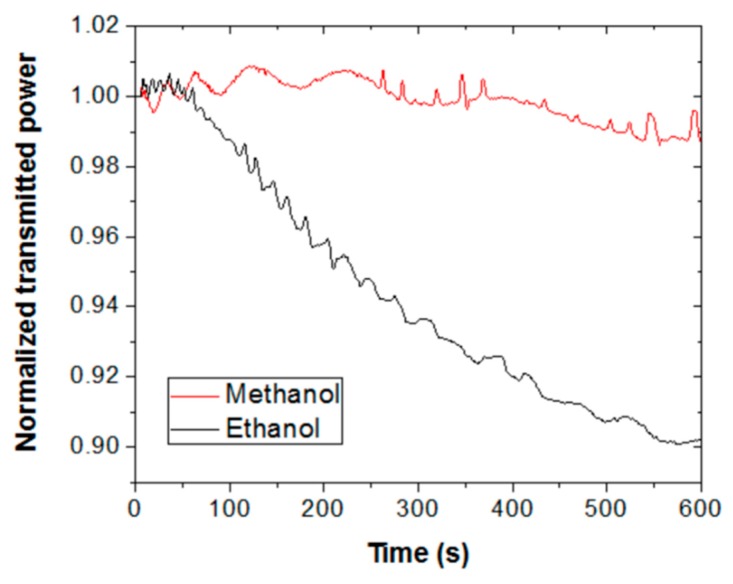
Scotch tape optical response for methanol and ethanol for an exposure time of 10 min. Saturation signal of ethanol is greater than that of methanol.

**Figure 6:**
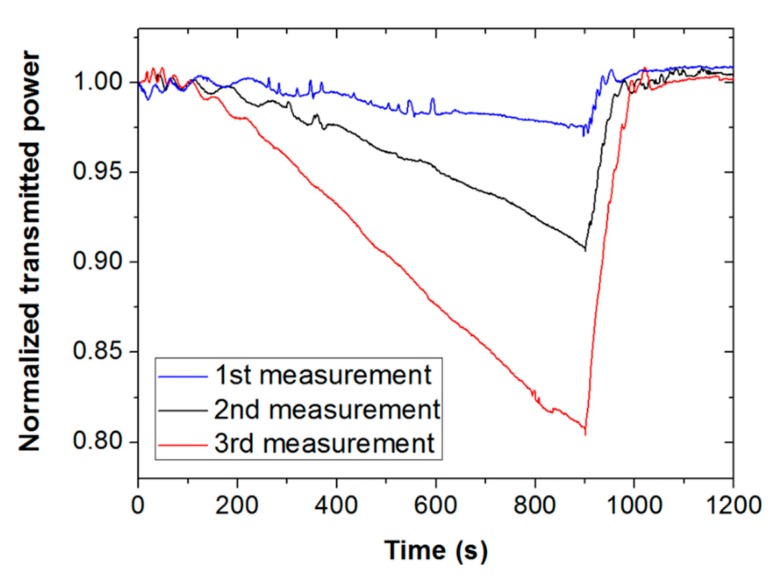
Optical response of a piece of Scotch tape when subjected to three consecutive exposure (0–900 s)–exhaust (900–1200 s) measurements of methanol vapor. All signals differ for an exposure time of 15 min (900 s).

Figure 5 shows the sensor response for ethanol (black line) and methanol (red line) vapors for an exposure time of 10 min. Note that the saturation signal for ethanol is greater than that for methanol, supporting that the solubility parameter of the responsive polymer is closer to that of ethanol. Thus, saturation signal could be also used as a parameter to differentiate both alcohols. However, it should be mentioned that exposure times longer than 5 min (in particular, 10 min and 15 min) led to non-repetitive results for both ethanol and methanol. Figure 6 illustrates this fact by showing three consecutive measurements of methanol vapor for an exposure time of 15 min. It is seen for all measurements that when the tape is exposed for such a long time, light attenuation starts increasing with exposure time. All signals recover well when the vapor in the cuvette is removed. However, the second and third measures clearly deviate from the previous measurement during vapor exposure, exhibiting higher attenuation. This suggests some permanent or long-term modification of the Scotch tape material due to prolonged vapor contact, which prevented the device from being operated in saturated vapor conditions.

**Figure 7:**
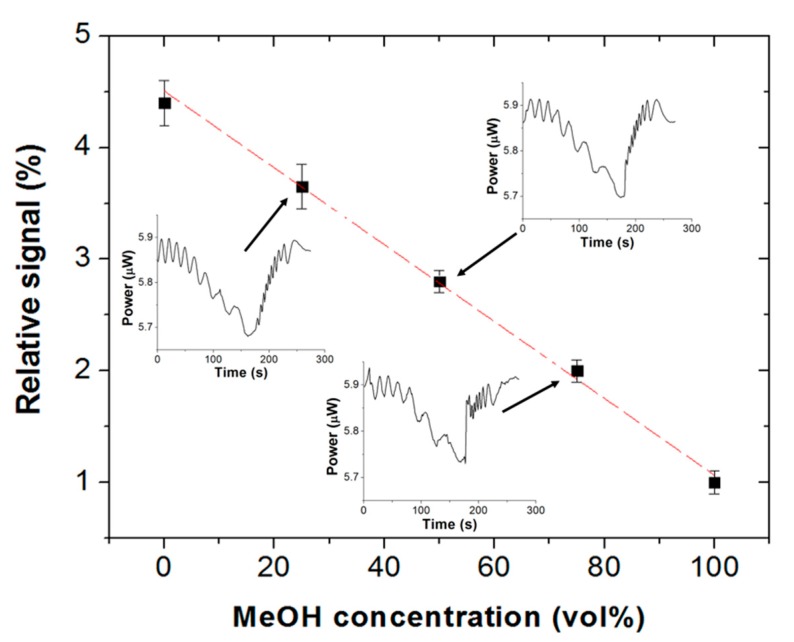
Relative signal of a Scotch tape vapor sensor as a function of liquid methanol concentration in ethanol–methanol liquid mixtures. Vapor exposure time = 3 min. Measured data points are fitted by a linear function (adjusted R^2^ = 0.995). Inset: Graphs illustrating representative measurements for 25%, 50%, and 75% methanol concentrations.

Sensor calibration was carried out by exposing the Scotch tape to vapors from ethanol–methanol liquid mixtures, ranging from pure ethanol to pure methanol. Both alcohols have similar natures (molecular structure and properties), and the corresponding binary mixture can be considered as nearly ideal [20]. That is, both solvents evaporate simultaneously at different rates and have little effect upon each other [21]. Figure 7 plots the relative signal of a Scotch tape vapor sensor, defined as [(P_1_ − P_2_)/P_1_] × 100, where P_1_ and P_2_ are the transmitted optical powers at 635 nm wavelength at the beginning and at the end, respectively, of the vapor exposure interval (3 min) as a function of the volume fraction of liquid methanol. The sensor exhibits a linear response (adjusted R^2^ = 0.995) from 0 vol% to 100 vol% methanol, with a sensitivity of S = −0.034%/(vol%). The limits of detection (LOD) of the sensor (calculated as LOD = |3σ/S|, where σ is the standard deviation of the baseline) equal 17.6 vol% liquid methanol and 8.8 vol% liquid ethanol. For the sake of comparison, LODs reported for enzymatic biosensors [6] were 3 vol% liquid methanol and 23 vol% liquid ethanol, and 0.5–2.1 vol% liquid ethanol for fluorescence probes [9].

**Figure 8:**
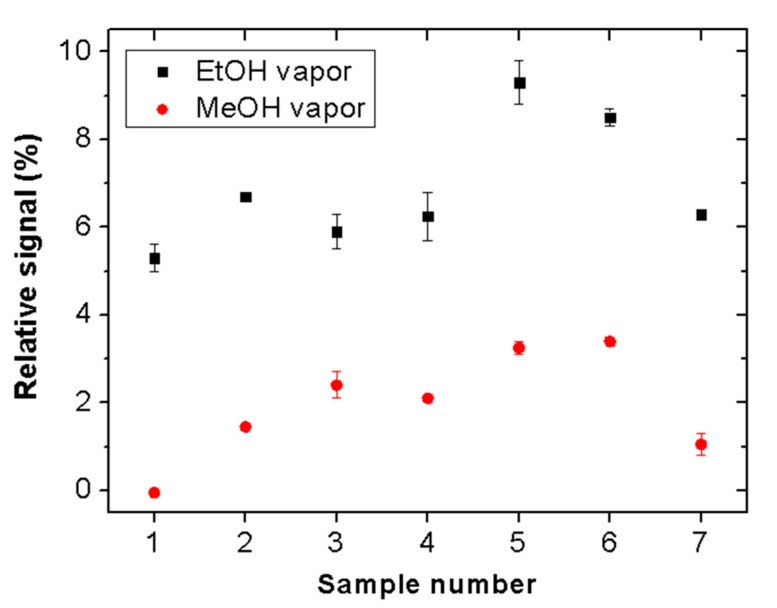
Relative response variation of seven different pieces of Scotch tape after 5 min exposure to ethanol (black square dots) and methanol (red circular dots) vapors. All tested samples exhibit clear selectivity for ethanol vapor.

The reproducibility of the proposed sensor was tested by measuring the responses of seven different pieces of the considered Scotch tape. Figure 8 shows the relative sensor signals for ethanol and methanol (vapor exposure time = 5 min). All tested samples exhibit clear selectivity for ethanol vapor. The signal magnitudes vary in the ranges of 5% to 9% and 0% to 3.5% for ethanol and methanol, respectively. These variations may be attributed to thickness and morphological inhomogeneities of the adhesive material of the tape.

**Figure 9:**
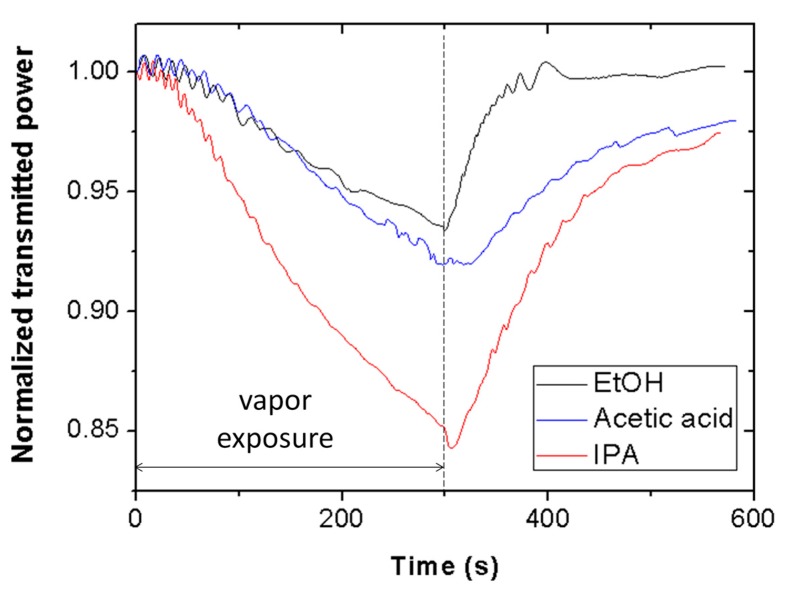
Transmitted optical power at 635 nm wavelength through a piece of Scotch tape subjected to ethanol (EtOH) (black line), acetic acid (blue line), and isopropanol (IPA) (red line) vapor exposure.

Study of the sensor sensitivity to other VOCs provides information concerning interferents and potential applications of the Scotch tape sensor in multianalyte detection (e.g., odor sensors). Figure 9 shows the transmitted power at 635 nm wavelength through a piece of Scotch tape as a function of time for ethanol (black line), acetic acid (blue line), and isopropanol (red line) vapors. Exposure time is equal to 5 min. As carried out for ethanol and methanol, the effect of isopropanol and acetic acid vapor exposure on the inner surface of the interrogation cuvette was examined, resulting in negligible contribution of the cuvette to the measured signals. Figure 9 reveals that the tape is sensitive to acetic acid and isopropanol vapors, exhibiting distinct sensitivities for each VOC. The selectivity of the sensor, defined as the ratio of the sensor signal for methanol and that for other VOC at a vapor exposure time of 5 min, is 1.06, 1.09, and 1.16 for ethanol, acetic acid, and isopropanol, respectively. It is also observed that the three signals recover at different rates. Thus, both signal amplitude and recovery time could be used as differentiation elements to discriminate these VOCs with the proposed Scotch tape optical vapor sensor.

Finally, it should be mentioned that the chosen distance between the tape and the photodetector (55 cm) was determined and limited by the length of the characterization optical table. A longer distance would lead to larger sensor signal variations, since the photodetector would collect less scattered light. However, a compact system is desirable, particularly for on-the-spot applications; for this, the addition of optical components (lenses, collimators, mirrors) could help to reduce the sensor system dimensions.

## 4. Conclusions

It has been demonstrated that conventional Scotch tape can reliably discriminate ethanol from methanol by simple optical means. This, together with its adhesive properties and low cost, make the studied Scotch tape a suitable disposable sensing element for easy integration in optical platforms targeting ethanol–methanol mixture testing, either on-the-spot or remotely. This is a proof-of-concept work; future investigations will be directed towards studying other types of adhesive tape, the influence of environmental conditions (temperature and relative humidity) on the sensor performance, and the optimization of the optical interrogation set-up for increasing sensitivity and compactness.
